# Holistic Management of Obesity in Patients with Incidental Cancer After Unprovoked Deep Vein Thrombosis: A Cardiometabolic and Antithrombotic Framework

**DOI:** 10.3390/cancers18142212

**Published:** 2026-07-09

**Authors:** Calogero Geraci, Rossella Cannarella, Valentina Morello, Valentina Paternò, Salvatore Massimo Petrina, Roberta Esposito, Giulio Geraci, Rosita A. Condorelli, Sandro La Vignera, Aldo E. Calogero

**Affiliations:** 1 Cardio Obesity Group, ACRIS ETS APS—Associazione Cuore Rene Ipertensione Sicilia, 93017 Caltanissetta, Italy; 2 Department of Clinical and Experimental Medicine, University of Catania, 95124 Catania, Italyaldo.calogero@unict.it (A.E.C.); 3 Cardiology Unit, Giovanni Paolo II Hospital, 97100 Ragusa, Italy; 4 Department of Translational Medical Sciences, University of Naples Federico II, 80131 Naples, Italy; 5 University of Unikore, 94100 Enna, Italy

**Keywords:** obesity, unprovoked deep vein thrombosis, occult cancer, cancer-associated thrombosis, GLP-1 receptor agonists, tirzepatide, semaglutide, SGLT2 inhibitors, apixaban, CKD-EPI, cancer cachexia, multidisciplinary management

## Abstract

**Background:** Obesity has evolved from a morphometric descriptor to a chronic, relapsing, multisystem disease in which low-grade adipose-tissue inflammation, insulin resistance, endothelial dysfunction, and a prothrombotic, pro-tumorigenic milieu coexist. Venous thromboembolism (VTE) and malignancy share this substrate through a well-established bidirectional link: within twelve months of an unprovoked deep vein thrombosis (DVT), 6–15% of patients are diagnosed with occult cancer, a substantial proportion of which are already locally advanced or metastatic at detection. In this context, obesity acts as a dual amplifier of both thrombotic and oncologic risk, making the post-DVT patient with an incidentally diagnosed cancer a paradigmatic candidate for integrated management. **Objective:** To propose an evidence-based, unifying cardiometabolic and antithrombotic clinical framework that reconciles (i) risk-stratified screening for occult malignancy after unprovoked DVT, (ii) contemporary pharmacotherapy of obesity, and (iii) cancer-associated thrombosis (CAT) management, in the light of evidence published through 2025. **Methods:** Narrative synthesis based on a structured literature search (PubMed/MEDLINE, Embase, Scopus; 2000–March 2025) of current guidelines (ISTH, ASH, ESC Cardio-Oncology, EASO) and pivotal randomized evidence on GLP-1 and dual GLP-1/GIP receptor agonists (semaglutide, tirzepatide), SGLT2 inhibitors (empagliflozin, dapagliflozin), and direct oral anticoagulants (DOACs), with critical interpretation of recent meta-analytic data on oncologic signals, cardiovascular outcomes, and bleeding safety. **Results:** Three evidence-based pillars emerge: (1) limited screening complemented by age- and sex-specific testing in patients ≥ 50 years with unprovoked DVT, enhanced by FDG-PET/CT for high-risk phenotypes in which obesity itself degrades the sensitivity of clinical examination and conventional imaging; (2) GLP-1/GIP receptor agonist-based anti-obesity pharmacotherapy, associated in observational cohorts with a 17% relative reduction in overall cancer incidence (HR 0.83; 95% CI 0.76–0.91) and neutral-to-reassuring oncologic signals, but requiring cautious, case-by-case use in patients with active cancer because of the risk of accelerating sarcopenia and cachexia; and (3) apixaban as the preferred DOAC for cancer-associated thrombosis, with a superior efficacy-to-bleeding ratio in network meta-analyses and a reduced-dose extended strategy now validated by the API-CAT trial. **Conclusions:** Patients with obesity presenting with unprovoked DVT and an incidentally detected cancer embody a cardiometabolic–antithrombotic continuum. A framework integrating structured occult-cancer screening, judicious obesity pharmacotherapy, and apixaban-preferred anticoagulation—coordinated by a multidisciplinary team and illustrated here by a case-based pathway—offers the most rational, evidence-aligned approach. Prospective, dedicated trials in this specific phenotype are urgently warranted.

## Simple Summary

Obesity, blood clots, and cancer are closely connected through common biological mechanisms such as chronic inflammation, insulin resistance, and increased blood clotting. Patients who develop an unprovoked deep vein thrombosis (DVT) have an increased risk of being diagnosed with an undetected cancer, and obesity further increases both thrombotic and cancer risk. This review proposes a practical, multidisciplinary approach for managing these patients. It discusses how to identify occult cancer after unprovoked DVT, the potential role and limitations of modern anti-obesity medications such as GLP-1 receptor agonists and tirzepatide, the cardiorenal benefits of SGLT2 inhibitors, and the optimal use of anticoagulant therapy, with a preference for apixaban in most patients with cancer-associated thrombosis. Because weight-loss medications may worsen muscle loss in patients with active cancer, they should be used only after careful nutritional and oncological assessment. Overall, this review highlights the importance of coordinated care involving multiple specialists to improve outcomes in patients at the intersection of obesity, thrombosis, and cancer.

## 1. Introduction

The co-occurrence of obesity, venous thromboembolism (VTE), and malignancy in the same patient is no longer a clinical curiosity but a predictable convergence driven by shared pathophysiological substrates. Obesity—defined by the World Health Organization (WHO) as a body mass index (BMI) ≥ 30 kg/m^2^—has become the most common form of malnutrition worldwide: the NCD Risk Factor Collaboration estimates that more than one billion people were living with obesity in 2022, comprising nearly 880 million adults and 159 million children and adolescents, with the age-standardized prevalence having more than doubled in adults since 1990 [1]. Simultaneously, venous thromboembolic disease affects approximately 1–2 per 1000 individuals annually in Western populations, and malignancy is among the most readily identifiable attributable causes in provoked cases. The intersection of these three conditions in a single patient represents a diagnostic and therapeutic challenge that no single specialty can address in isolation.

Importantly, the link between excess adiposity and cancer is not merely associative. Several large cohort and meta-analytic studies demonstrate a higher incidence of site-specific cancers—notably breast, colorectal, endometrial, and pancreatic—in individuals with obesity compared with normal-weight individuals, with risk gradients that track with the degree and duration of adiposity [2,3]. This epidemiological signal provides the clinical anchor for considering obesity an active driver, rather than a bystander, of oncologic risk in the post-DVT patient.

Obesity has undergone a fundamental reconceptualization over the past two decades. The European Association for the Study of Obesity (EASO) and contemporary clinical practice guidelines formally recognize obesity as a chronic, relapsing, multisystem disease, not merely an anthropometric index [4]. This reclassification shifts the therapeutic mandate from symptomatic weight reduction to disease modification, requiring long-term pharmacological and behavioral strategies analogous to those used in hypertension, diabetes mellitus, or heart failure. Within this disease model, adipose tissue is reconceived not as an inert energy reservoir but as a complex endocrine organ capable of sustaining systemic low-grade inflammation, hypercoagulability, and mitogenic signaling [2,5].

The relationship between VTE and cancer is bidirectional and quantitatively significant. Venous thrombosis was first recognized as a paraneoplastic phenomenon by Armand Trousseau in 1865, and subsequent observational data have solidified this link across large epidemiological cohorts. Among patients presenting with a first unprovoked proximal DVT or pulmonary embolism, 6–15% receive a new cancer diagnosis within the subsequent twelve months, and a substantial proportion of these neoplasms are already locally advanced or metastatic at detection [6,7,8]. This high-stakes scenario obligates the treating physician to move beyond thrombosis management and engage systematically with malignancy screening, a task complicated by the confounding clinical and biochemical features that obesity introduces [9,10].

**Figure 1:**
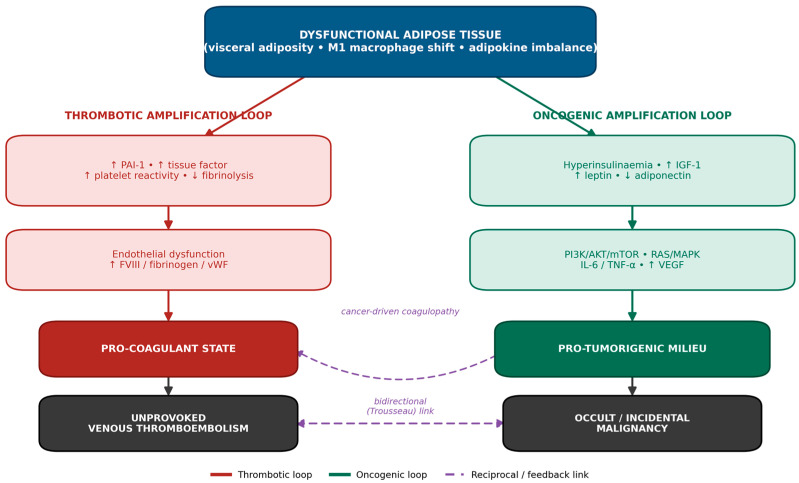
Obesity as a dual amplifier of thrombotic and oncogenic risk. Dysfunctional adipose tissue feeds two reciprocally reinforcing loops. The thrombotic loop (left)—↑ PAI-1, ↑ tissue factor, ↑ platelet hyperreactivity, ↓ impaired fibrinolysis, and endothelial dysfunction—generates a pro-coagulant state predisposing to unprovoked venous thromboembolism. The oncogenic loop (right)—hyperinsulinaemia, ↑ IGF-1, ↑ leptin/↓ adiponectin imbalance, PI3K/AKT/mTOR and RAS/MAPK activation, and ↑ VEGF—generates a pro-tumorigenic milieu predisposing to occult malignancy. The bidirectional Trousseau link and cancer-driven coagulopathy (dashed) close the system. IGF-1, insulin-like growth factor-1; PAI-1, plasminogen activator inhibitor-1; vWF, von Willebrand factor; VEGF, vascular endothelial growth factor.

Obesity amplifies both arms of the VTE–cancer dyad. On the thrombotic side, it promotes endothelial dysfunction, platelet hyperreactivity, tissue factor (TF) overexpression, elevated plasminogen activator inhibitor-1 (PAI-1), and reduced fibrinolytic activity, collectively creating a persistently pro-coagulant milieu [4,11]. On the oncogenic side, adipose-tissue dysfunction drives hyperinsulinemia, elevation of insulin-like growth factor-1 (IGF-1), leptin excess, adiponectin deficiency, and chronic interleukin-6 (IL-6)/tumor necrosis factor-alpha (TNF-α)-mediated inflammation—each of which activates canonical oncogenic cascades including phosphatidylinositol 3-kinase (PI3K)/AKT/mechanistic target of rapamycin (mTOR) and RAS/mitogen-activated protein kinase (MAPK) [3,12,13]. The result is a biological environment that simultaneously lowers the threshold for venous clot formation and accelerates malignant transformation. Obesity thus functions as a “dual amplifier,” and the patient who presents with an unprovoked DVT and is subsequently found to harbor an incidental neoplasm occupies a uniquely vulnerable position along this continuum (Figure 1).

The advent of two pharmacological revolutions—glucagon-like peptide-1 (GLP-1) receptor agonists and direct oral anticoagulants (DOACs)—has created, for the first time, a set of clinical tools capable of addressing multiple nodes of this cardiometabolic–antithrombotic continuum simultaneously. This narrative review pursues three integrated objectives: first, to appraise the evidence for structured occult-cancer screening following unprovoked DVT, with specific attention to the high-risk obese phenotype; second, to critically evaluate the cardiometabolic and oncologic profiles of GLP-1/GIP receptor agonists and SGLT2 inhibitors in this population, including the specific caveats raised by active malignancy; and third, to synthesize the evidence on DOAC-based anticoagulation for CAT, with particular attention to apixaban across BMI strata. The ultimate aim is to propose a holistic, multidisciplinary framework that is evidence-grounded, practically implementable through a case-based pathway, and ready for prospective validation.

### Literature Search Strategy

This narrative review was informed by a structured search of PubMed/MEDLINE, Embase, and Scopus for records published between January 2000 and March 2025, supplemented by hand-searching of reference lists of key articles and of the most recent guideline documents. The search combined controlled vocabulary and free-text terms across three thematic blocks: (i) “obesity,” “visceral adiposity,” “adipokines,” “insulin resistance,” and “obesity-related cancer”; (ii) “unprovoked venous thromboembolism,” “deep vein thrombosis,” “occult cancer,” “cancer-associated thrombosis,” and “FDG-PET/CT”; and (iii) “GLP-1 receptor agonist,” “tirzepatide,” “semaglutide,” “SGLT2 inhibitor,” “direct oral anticoagulant,” and “apixaban.” Priority was given to randomized controlled trials and their primary publications, individual patient-data and network meta-analyses, and current society guidelines (ISTH, ASH, ESC Cardio-Oncology, EASO). Reviews were used to provide mechanistic context but were not substituted for primary trial citations. English-language human studies were included; case reports and conference abstracts without subsequent full publication were excluded. Where landmark trials are discussed, the original report is cited in preference to secondary syntheses.

## 2. The Oncobiological Milieu of Obesity: Shared Pathways with Thrombosis and Cancer

### 2.1. Adipose Tissue as an Active Endocrine Organ

The traditional view of white adipose tissue as a passive energy repository has been superseded by recognition of its role as a highly active endocrine, paracrine, and immune organ. Adipocytes and adipose-resident stromal cells secrete a diverse repertoire of adipokines that regulate systemic metabolic homeostasis, vascular tone, and immune surveillance [14]. In obesity, adipocytes undergo hypertrophy, the stromal vascular fraction expands, and macrophage infiltration shifts from an anti-inflammatory (M2) to a pro-inflammatory (M1) polarization state, producing chronic low-grade inflammation characterized by elevated TNF-α, IL-6, monocyte chemoattractant protein-1 (MCP-1), and C-reactive protein (CRP) [12,15].

Leptin, whose plasma levels are proportional to fat mass, exerts mitogenic, angiogenic, and anti-apoptotic effects through the JAK2/STAT3 pathway, and its receptors are expressed in breast, colorectal, and endometrial malignancies [14,16]. Conversely, adiponectin—which decreases as adiposity increases—functions as an insulin sensitizer with anti-proliferative, anti-inflammatory, and pro-apoptotic effects via AMPK and PPAR-α signaling. The leptin-to-adiponectin ratio, which rises with BMI, is an integrative biomarker of oncologic and metabolic risk [14]. Resistin, predominantly macrophage-derived in humans, contributes to insulin resistance and inflammatory signaling through NF-κB activation [16].

### 2.2. Insulin Resistance, Hyperinsulinemia, and the IGF-1 Axis

Obesity-driven insulin resistance leads to compensatory beta-cell hypersecretion and chronic hyperinsulinemia. Insulin and its functional analog IGF-1 activate the PI3K/AKT/mTOR and RAS/MAPK pathways through receptor tyrosine kinase signaling—among the most frequently dysregulated cascades in human cancers [3,16]. High circulating insulin reduces hepatic IGF-binding protein-1 and IGFBP-2, increasing bioavailable IGF-1, which promotes proliferation, inhibits apoptosis, and facilitates anchorage-independent growth. Epidemiological data link hyperinsulinemia to incident colorectal, breast, endometrial, and pancreatic cancers independent of BMI [3]. In the VTE context, insulin resistance also promotes PAI-1 transcription through the MAPK pathway, impairing fibrinolysis and favoring thrombus persistence [11].

### 2.3. Visceral Adiposity, Epicardial Fat, and Endothelial Dysfunction

Visceral adipose tissue (VAT) is metabolically distinct from subcutaneous fat and exhibits far greater inflammatory and endocrine activity. Excess VAT is associated with elevated free fatty acids, oxidative stress, endothelial nitric oxide synthase (eNOS) uncoupling, reduced bioavailability of nitric oxide (NO), and upregulation of endothelin-1 and angiotensinogen—collectively producing endothelial dysfunction with impaired vasodilation, increased vascular permeability, and enhanced leukocyte adhesion [4,11]. Epicardial fat, contiguous with the coronary vasculature, adds a paracrine layer of inflammatory mediators and may modulate coagulation through local TF and von Willebrand factor (vWF) expression. The metabolic syndrome has been independently associated with unprovoked VTE in patient-level meta-analyses [4].

### 2.4. Platelet Hyperreactivity, Tissue Factor Overexpression, and Impaired Fibrinolysis

The hemostatic system in obesity is tilted toward thrombogenesis across multiple nodes of the coagulation cascade. Platelet hyperreactivity is mediated in part by leptin-induced upregulation of surface glycoproteins and reduced intraplatelet NO [11]. TF, the primary initiator of extrinsic coagulation, is overexpressed in adipocyte membranes and shed into the circulation on microparticles from both adipocytes and tumor cells, amplifying thrombin generation in the cancer–obesity context [17]. PAI-1 is substantially elevated, particularly with VAT excess, impairing fibrinolysis and prolonging thrombus duration [11]. Factor VIII, fibrinogen, and vWF are also chronically elevated [4].

### 2.5. The Tumor Microenvironment Modulated by Adiposity

Cancer-associated adipocytes, which undergo delipidation and phenotypic transformation near tumor cells, supply energy substrates, cytokines, and remodeling enzymes to the developing tumor [15]. They release fatty acids—particularly oleic acid and palmitate—that adjacent malignant cells take up to fuel beta-oxidation, a symbiosis well characterized in breast, ovarian, and liposarcoma models. Adipose-derived stromal cells promote immunosuppressive microenvironments by favoring M2 polarization, regulatory T-cell expansion, and myeloid-derived suppressor cell accumulation [15]. The adipokine-rich microenvironment also promotes angiogenesis through upregulation of vascular endothelial growth factor (VEGF), worsening both tumor perfusion and the local pro-coagulant state [13].

### 2.6. Synthesis: Obesity as the Dual Amplifier

Taken together, the mechanisms above position obesity at the intersection of two converging amplification loops, summarized in Figure 1. A thrombotic loop—platelet hyperreactivity, TF overexpression, PAI-1 elevation, and endothelial dysfunction—raises the intrinsic probability of VTE. An oncogenic loop—hyperinsulinemia, IGF-1 excess, adipokine dysregulation, and chronic inflammation—programs a pro-tumorigenic microenvironment. The two loops are reciprocally reinforcing, and cancer, once present, further dysregulates hemostasis. When VTE occurs in this setting it is simultaneously a medical emergency and a symptomatic expression of the milieu that has been nurturing occult malignant disease. Rather than restate the mechanistic detail of Section 2.1, Section 2.2, Section 2.3, Section 2.4 and Section 2.5, Figure 1 provides the integrative schema that underpins the therapeutic strategy proposed in this review.

## 3. Occult Cancer Screening After Unprovoked DVT in the Obese Patient

The association between unprovoked VTE and occult malignancy is well established, and the principles of screening have been extensively codified in the general population; this section therefore focuses on the elements that are specific to, or modified by, obesity rather than re-deriving the general algorithm (Table 1).

### 3.1. Epidemiology and Risk Stratification

Across prospective cohorts and the individual patient-data meta-analysis of Van Es and colleagues, the 12-month cumulative incidence of a new cancer diagnosis after unprovoked VTE ranges from roughly 4% to 12%, rising to the upper end of the 6–15% range in higher-risk subgroups; a substantial fraction of these cancers are already advanced at detection, implying a window of diagnostic opportunity [6,7,8]. The most consistent predictors of occult malignancy are age ≥ 50 years, recurrent unprovoked VTE, special-site thrombosis (splanchnic, cerebral, bilateral pelvic, or upper-extremity), and a D-dimer disproportionate to the thrombotic burden—patients with a D-dimer > 4000 ng/mL show an approximately four-fold higher incidence of occult cancer than those with a D-dimer < 2000 ng/mL (adjusted HR 4.12, 95% CI 1.54–11.04) over a median follow-up of approximately 5.3 years [6,9]. Colorectal, lung, pancreatic, genitourinary, and hematologic malignancies predominate [6].

### 3.2. ISTH Limited Screening and the Role of FDG-PET/CT

International guidance (ISTH, ASH) recommends a tiered approach anchored by a universal “limited screening” strategy: clinical history, physical examination, a standard laboratory panel (CBC, LFTs, LDH, creatinine), chest radiography, and age- and sex-appropriate cancer screening according to national programs [9,31]. The SOME trial showed that adding routine thoraco-abdomino-pelvic CT to limited screening did not significantly reduce missed cancers (absolute difference 2%, *p* = 0.81) or mortality, establishing limited screening as the first-line standard and reserving extended imaging for higher-risk patients [9]. Whole-body FDG-PET/CT offers high negative predictive value (reported up to ~99%) and, in the MVTEP trial, a negative result identified patients significantly less likely to be subsequently diagnosed with cancer (1/186 vs. 9/193; *p* = 0.02); the ongoing MVTEP2 trial (NCT04304651) is testing a PET/CT-augmented strategy in patients ≥ 50 years with a first unprovoked VTE [18,32]. Beyond conventional markers, ctDNA, cell-free DNA methylation, soluble P-selectin, and NET-related markers remain investigational and are not recommended outside research protocols [6].

### 3.3. Why Obesity Lowers the Threshold for FDG-PET/CT

Obesity specifically degrades each tier of standard screening and provides the rationale for a more proactive imaging strategy in selected patients. First, physical examination is markedly less sensitive in patients with severe adiposity, reducing the reliability of palpatory detection of hepatomegaly, lymphadenopathy, or abdominal masses. Second, D-dimer correlates positively with adipose mass independently of thrombosis, so reference intervals derived from normal-weight populations may misclassify obese patients [10]. Third, conventional cross-sectional imaging in high-BMI individuals is prone to beam-hardening artifact and reduced contrast resolution. Taken together, these limitations justify proceeding to whole-body FDG-PET/CT in obese patients ≥ 50 years who carry one or more additional high-risk features—D-dimer > 4000 ng/mL, recurrent or special-site DVT, or unexplained constitutional symptoms—in whom the diagnostic yield is highest and the limitations of standard work-up most pronounced [6,10]. Awareness of physiological and brown-adipose FDG uptake is required for accurate interpretation in this population.

## 4. GLP-1 and Dual GLP-1/GIP Receptor Agonists at the Crossroads of Oncology

### 4.1. Mechanism of Action

GLP-1 is an incretin secreted postprandially by intestinal L-cells that acts through the GLP-1 receptor (GLP-1R), a class B G protein-coupled receptor coupled to adenylyl cyclase, potentiating glucose-dependent insulin secretion and suppressing glucagon. GLP-1R is also expressed in the hypothalamus, brainstem, vagal afferents, cardiac myocytes, renal tubular cells, vascular endothelium, and adipocytes, accounting for appetite suppression, delayed gastric emptying, natriuresis, reduced hepatic steatosis, and anti-inflammatory signaling [19,20]. Dual GLP-1/GIP receptor agonists (“twincretins”) additionally activate the glucose-dependent insulinotropic polypeptide receptor (GIPR), synergistically enhancing the anorexigenic and adipolytic effects of GLP-1R activation; the clinical consequence, exemplified by tirzepatide, is weight loss exceeding that of GLP-1R monoagonism, with mean reductions of 15–22% in pivotal trials [20].

### 4.2. Semaglutide: Cardiovascular and Metabolic Outcomes

Semaglutide, a long-acting GLP-1R agonist, achieved a 20% relative reduction in the primary composite MACE endpoint in the SELECT trial (HR 0.80; 95% CI 0.72–0.90; *p* < 0.001) among 17,604 adults with overweight or obesity and established cardiovascular disease but without diabetes [19]. SELECT established that GLP-1R agonism confers cardiovascular protection at least partly independent of glucose lowering, attributable to anti-inflammatory and anti-atherogenic mechanisms; high-sensitivity CRP fell by approximately 40% relative to placebo, a magnitude of anti-inflammatory effect with plausible oncologic relevance [19].

### 4.3. Tirzepatide: SURMOUNT and SURPASS Programs

Tirzepatide, the first approved dual GIP/GLP-1 receptor agonist, produced a mean weight reduction of 20.9% (15 mg dose) in the SURMOUNT-1 trial of 2539 adults with obesity or overweight without diabetes, with ≥20% weight loss in 57% of participants [20]. Within the SURPASS glycometabolic program, the head-to-head SURPASS-2 trial demonstrated superiority of tirzepatide over semaglutide 1 mg for both HbA1c and body-weight reduction in type 2 diabetes [21]; subsequent reports and real-world data confirm reductions in systolic blood pressure, triglycerides, and markers of hepatic steatosis. For the obese patient with post-DVT incidental cancer, the therapeutic potential extends beyond weight loss to visceral-fat redistribution, insulin-sensitivity improvement, adipokine normalization, and reduction in systemic inflammatory biomarkers.

### 4.4. Oncologic Signals: Evidence Synthesis and Critical Appraisal

The oncologic profile of GLP-1/GIP receptor agonists is among the most actively researched topics in cardiometabolic medicine, informed by three principal sources. First, a 2025 retrospective cohort in JAMA Oncology of 86,632 adults with obesity or overweight found that GLP-1RA use was associated with a 17% relative reduction in overall cancer incidence (HR 0.83; 95% CI 0.76–0.91; *p* < 0.001) over a median ~3.5 years, with significant site-specific reductions for endometrial (HR 0.75), ovarian (HR 0.53), and meningioma (HR 0.69), and a non-significant signal for kidney cancer (HR 1.38; 95% CI 0.99–1.93) warranting surveillance [22]. Second, a 2025–2026 systematic review and meta-analysis of 48 RCTs (94,245 participants) in the Annals of Internal Medicine concluded that GLP-1RAs probably have little or no effect on thyroid, pancreatic, breast, or kidney cancer risk (all moderate-certainty), consistently across semaglutide and tirzepatide subgroups [33].

Third, a 2024 TriNetX cohort in Cancers of more than 1.1 million patients reported reduced hazard ratios across several individual gastrointestinal cancer types (pooled HR 0.67), breast cancer (HR 0.72), and prostate cancer (HR 0.68), with semaglutide showing particularly strong protection for colorectal cancer (HR 0.45) [34]. To avoid ambiguity, “multiple GI cancers” here denotes several distinct gastrointestinal primary cancer types analyzed separately (e.g., colorectal, gastric, esophageal, pancreatic, hepatobiliary), not multiple synchronous primaries or multifocal metastatic disease within one patient. The magnitude of these associations likely reflects differences in follow-up and population characteristics and should be interpreted with caution regarding residual confounding.

### 4.5. Thyroid Cancer: Pharmacovigilance Context

The class boxed warning regarding medullary thyroid carcinoma (MTC) and multiple endocrine neoplasia type 2 (MEN2) derives from rodent C-cell carcinogenesis studies. GLP-1 receptors are expressed at high density on rodent thyroid C-cells but at substantially lower levels in humans, making interspecies extrapolation uncertain. Large multinational pharmacoepidemiological studies have not demonstrated a significant increase in thyroid cancer incidence over follow-up of up to ~4.5 years [33]. The practical implication is that GLP-1RA use is restricted in patients with personal or first-degree family history of MTC or MEN2 but need not be withheld from appropriately screened patients without these risk factors.

### 4.6. Anti-Inflammatory and Antitumorigenic Mechanisms

Several mechanisms beyond weight loss make an oncologic benefit biologically plausible. GLP-1R agonism suppresses the NLRP3 inflammasome and lowers CRP and IL-6 (SELECT: CRP ↓ ~40%) [19]. The disproportionate reduction in visceral adipose tissue directly attenuates the adipokine-mediated signaling described in Section 2. Improved insulin sensitivity reduces bioavailable IGF-1 and thereby PI3K/AKT/mTOR and RAS/MAPK stimulation in transformed cells. GLP-1R is also expressed on colorectal, pancreatic, and endometrial cancer cell lines, where activation may trigger growth-inhibitory cascades via PKA-mediated CREB phosphorylation and β-catenin destabilization [34].

### 4.7. GLP-1/GIP Therapy in Active Cancer: Sarcopenia, Cachexia, and Timing

A central caveat—and a point on which we have deliberately tempered our earlier position—concerns the use of these agents in patients with active malignancy. The weight loss induced by GLP-1/GIP receptor agonists is not exclusively adipose: a variable proportion is lean mass. In a patient with cancer, this is superimposed on disease- and treatment-related anorexia, the catabolic drive of systemic inflammation, and frequently anorexigenic chemotherapy, creating a real risk of accelerating sarcopenia and cancer cachexia, eroding performance status, and compromising chemotherapy tolerance. Initiating potent anorexigenic therapy at, or immediately after, the diagnosis of an incidental cancer is therefore not advisable as a default.

Accordingly, in patients with active cancer these agents should be considered case by case rather than started immediately, and only after a careful nutritional assessment (dietitian/clinical nutritionist), characterization of disease stage, and clarification of treatment intent (curative vs. palliative). In practice, this approach should be restricted to carefully selected patients without overt or impending cancer cachexia, and preferably initiated within a structured multidisciplinary setting once the oncologic picture has stabilized, with longitudinal monitoring of body composition, muscle strength, and nutritional status. The strongest rationale for obesity pharmacotherapy in this population remains in patients in oncologic remission or with indolent disease and a favorable nutritional reserve.

### 4.8. Renal Function: Use in Advanced CKD and Dialysis

Renal considerations are relevant chiefly for safety monitoring rather than efficacy. Neither semaglutide nor tirzepatide requires dose adjustment in mild-to-moderate renal impairment, and pharmacokinetics are not meaningfully altered across eGFR strata. For severe impairment (eGFR < 3 0 mL/min/1.73 m^2^) and end-stage kidney disease including dialysis, the product labels indicate no mandatory dose adjustment but advise caution because clinical experience is limited; the main hazard is indirect—gastrointestinal adverse effects causing dehydration and pre-renal acute kidney injury—so gradual titration, antiemetic support, and attention to volume status are warranted, and initiation in this setting should be individualized [19,20,21]. These agents are not a substitute for renal replacement decisions and should be coordinated with nephrology when advanced CKD coexists.

## 5. SGLT2 Inhibitors: Cardio- and Nephroprotection in the Obese Oncologic Patient

### 5.1. Rationale and Position Within the Obesity–VTE–Cancer Triad

Sodium–glucose cotransporter-2 (SGLT2) inhibitors are introduced here not as antineoplastic agents but as an organ-protective therapy that addresses two vulnerabilities specific to the obese patient who develops cancer-associated thrombosis: the cardiotoxicity of several oncologic treatments and the renal jeopardy of obesity-related glomerular hyperfiltration compounded by nephrotoxic chemotherapy and contrast imaging. In this framework their role is complementary to anticoagulation and to obesity pharmacotherapy, providing a cardiorenal substrate on which the antithrombotic and metabolic strategies can be safely deployed.

### 5.2. Cardiovascular and Renal Outcome Evidence

The pivotal evidence derives from the original randomized cardiovascular and renal outcome trials rather than from secondary reviews. EMPA-REG OUTCOME randomized 7020 patients with type 2 diabetes and established cardiovascular disease to empagliflozin or placebo and demonstrated a significant reduction in cardiovascular death and in hospitalization for heart failure [23]. DAPA-HF extended the benefit to 4744 patients with heart failure and reduced ejection fraction—with or without diabetes—showing a reduction in the composite of worsening heart failure or cardiovascular death (HR 0.74; 95% CI 0.65–0.85) [24]. DAPA-CKD randomized 4304 patients with chronic kidney disease, again irrespective of diabetes status, and was stopped early for efficacy after dapagliflozin reduced the composite of sustained eGFR decline, end-stage kidney disease, or renal/cardiovascular death (HR 0.61; 95% CI 0.51–0.72) [25]. The consistency of cardiac and renal protection across diabetic and non-diabetic populations is what makes this class relevant to the obese oncologic patient independent of glycemic indication.

### 5.3. Relevance During Cancer Therapy

Anthracyclines, HER2-targeted agents, vascular endothelial growth factor (VEGF) pathway inhibitors, and several tyrosine-kinase inhibitors carry recognized cardiotoxic potential, and the 2022 ESC cardio-oncology guidelines frame baseline cardiovascular optimization as integral to oncologic care [35]. By reducing heart-failure events and stabilizing renal function, SGLT2 inhibition offers a plausible cardioprotective adjunct during potentially cardiotoxic regimens; preclinical and early clinical signals of direct anti-inflammatory and metabolic effects on the tumor–host interface remain hypothesis-generating and are deliberately not advanced here as a treatment indication [36,37,38]. The nephroprotective effect is of particular value in obesity, where glomerular hyperfiltration coexists with the cumulative renal insults of cancer therapy.

### 5.4. Safety Integration with Anticoagulation and Incretin Therapy

SGLT2 inhibitors do not have clinically meaningful pharmacokinetic interactions with direct oral anticoagulants, and their combination with GLP-1/GIP receptor agonists is mechanistically complementary. The principal cautions are euglycemic ketoacidosis—relevant during the reduced caloric intake of active cancer and around surgery, when temporary interruption is advised—volume depletion in patients already at risk of pre-renal injury, and genitourinary infection in immunosuppressed patients. Within the proposed pathway, SGLT2 inhibition is therefore introduced once the acute thrombotic and oncologic situation is stabilized and is monitored jointly by the treating team.

## 6. Anticoagulation for Cancer-Associated Thrombosis in Obesity

### 6.1. From Unprovoked VTE to Cancer-Associated Thrombosis

Chronologically, therapeutic anticoagulation begins at the moment the deep vein thrombosis is diagnosed, well before any occult cancer is identified. The subsequent discovery of malignancy does not initiate anticoagulation; it re-frames an already-running treatment as cancer-associated thrombosis (CAT), changing the preferred agent, the expected duration, and the intensity of follow-up. Recognizing this sequence avoids the conceptual error of treating cancer diagnosis as the starting point of the antithrombotic pathway.

### 6.2. Choice of Agent: DOACs vs. LMWH

Direct oral anticoagulants—apixaban in particular—are now first-line for most patients with CAT. The CARAVAGGIO trial randomized 1170 patients with cancer-associated VTE to oral apixaban or subcutaneous dalteparin and showed non-inferiority for recurrent VTE (HR 0.63; 95% CI 0.37–1.07) without a significant increase in major bleeding, including no excess of major gastrointestinal bleeding [28]. Apixaban is consequently attractive in obesity because it spares patients weight-based subcutaneous injections and avoids the dosing uncertainty of low-molecular-weight heparin (LMWH) at the extremes of body weight. Low-molecular-weight heparin retains a role when oral absorption is unreliable, in luminal gastrointestinal or genitourinary tumors at high mucosal bleeding risk, and in severe renal impairment where DOAC data are limited.

### 6.3. LMWH Dosing Uncertainty in Severe Obesity

In patients with severe obesity—particularly above approximately 120–150 kg—there is no consensus on whether therapeutic LMWH should be dosed strictly by actual body weight or empirically capped, and the pharmacodynamic reliability of fixed-cap regimens is unproven. Anti-Xa monitoring is often invoked in this setting but is poorly standardized and inconsistently predictive of clinical outcomes. This uncertainty is itself an argument for preferring a fixed-dose DOAC such as apixaban, for which the available CAT and obesity data are reassuring, rather than navigating un-validated weight-capped heparin schemes.

### 6.4. Drug–Drug Interactions with Anticancer Therapy

Apixaban is a substrate of CYP3A4 and P-glycoprotein (P-gp), so concurrent anticancer agents that strongly modulate these pathways can alter its exposure and must be reviewed at every regimen change (Table 2). Strong dual inducers reduce apixaban levels and increase thrombotic risk, whereas strong dual inhibitors raise levels and bleeding risk; in addition, agents that injure the mucosa or act on the vasculature can amplify bleeding independently of pharmacokinetics. Explicit examples make this practical: among inducers, the androgen-receptor pathway inhibitors enzalutamide and apalutamide, and the commonly co-administered corticosteroid dexamethasone, lower apixaban exposure; among inhibitors, the azole antifungals itraconazole and posaconazole, the protease inhibitor ritonavir, and the PI3K-delta inhibitor idelalisib raise it; and antiangiogenic agents such as bevacizumab increase mucosal bleeding risk through a non-pharmacokinetic mechanism.

### 6.5. Duration of Anticoagulation

The duration of anticoagulation in CAT is not “indefinite” in the abstract. Treatment should be continued as long as the cancer remains active and/or under treatment, with periodic re-assessment of the benefit–risk balance as the oncologic situation, renal function, platelet count, and bleeding risk evolve. Decisions to extend, reduce, or stop are revisited at defined intervals rather than fixed once at the outset.

### 6.6. Reduced-Dose Maintenance: The API-CAT Evidence

The question of intensity during prolonged maintenance has now been addressed directly. The API-CAT trial randomized 1766 patients with active cancer who had already completed at least six months of anticoagulation for VTE to reduced-dose apixaban 2.5 mg twice daily versus full-dose 5 mg twice daily for a further twelve months; reduced-dose apixaban was non-inferior for recurrent VTE while significantly reducing clinically relevant bleeding [30]. This supports a de-escalation strategy in the extended phase for selected patients with stable disease, complementing rather than replacing the full-dose initial treatment established by CARAVAGGIO. A caveat specific to the present framework must, however, be emphasized. Patients at the extremes of body weight—in particular those with severe obesity (BMI ≥ 40 kg/m^2^ or body weight > 120 kg)—were substantially underrepresented in the pivotal extended-phase CAT trials: in API-CAT they formed only a small subgroup (BMI ≥ 35 kg/m^2^) underpowered for definitive efficacy or safety conclusions, and other reduced-dose secondary-prophylaxis trials (e.g., RENOVE) reported no dedicated data for the severely obese. Because obesity itself raises both baseline and recurrent VTE risk, the de-escalation strategy validated by API-CAT should be extrapolated to the severely obese patient with caution and on an individualized basis; where uncertainty persists, continuation of full-dose apixaban—consistent with ISTH 2021 guidance favoring standard-dose DOACs for VTE in patients with BMI > 40 kg/m^2^ or weight > 120 kg—remains a reasonable default pending dedicated evidence.

### 6.7. Renal Function Estimation in Obesity

Accurate renal assessment is central to safe DOAC dosing, and in obesity the choice of equation matters. The Cockcroft–Gault formula, when populated with actual body weight, systematically overestimates creatinine clearance in obese patients and can mask clinically relevant impairment; CKD-EPI is therefore preferred for estimating renal function in this population, with Cockcroft–Gault reserved for explicit label-based dose decisions and interpreted with awareness of its weight dependence [39]. This distinction is not academic: overestimation of renal function can lead to inappropriately high anticoagulant exposure and avoidable bleeding in precisely the patients in whom CAT management is already most delicate.

### 6.8. Thrombophilia Testing and Who Manages Anticoagulation

In a patient with active cancer, the malignancy itself is a dominant, sufficient explanation for the prothrombotic state, and inherited thrombophilia workup is of negligible clinical utility because it almost never changes the decision to anticoagulate or the choice of agent; it should generally be omitted and considered only in rare, explicitly justified situations such as a strong personal and family history suggesting a high-risk hereditary disorder relevant to relatives. Likewise, the responsibility for managing anticoagulation should not be ascribed to any single specialty as a universal rule. Whether the treating physician is an internist, hematologist, oncologist, angiologist, or cardiologist varies substantially by national and local health-system organization, and the operational point is that anticoagulation is directed by a clearly identified treating physician within a multidisciplinary team rather than by a fixed discipline.

## 7. An Integrated, Case-Based Management Framework

### 7.1. Convergence of the Four Domains

The preceding sections describe four domains—occult-cancer screening, obesity pharmacotherapy, cardiorenal protection, and anticoagulation—that intersect in a single recognizable patient: the individual with obesity who presents with unprovoked DVT and is found to harbor an incidental malignancy. The value of treating them together rather than in parallel silos lies in sequencing and mutual constraint, where each decision is conditioned by the others rather than optimized in isolation. Table 1 distills the pivotal evidence underpinning each domain and translates it into the corresponding implication for the proposed framework.

### 7.2. Roles Within the Multidisciplinary Team

Coordinated care draws on vascular medicine or the relevant anticoagulation-managing discipline, oncology, cardiology, nephrology, clinical nutrition, and nuclear medicine. As noted in Section 6, the figure who manages anticoagulation depends on national and institutional organization; the framework therefore specifies functions—who estimates renal function, who screens for occult cancer, who decides anticoagulant agent and duration, who assesses nutritional reserve before any incretin therapy—rather than mandating a particular specialty for each task. Obesity pharmacotherapy is explicitly gated behind a nutritional and oncologic assessment rather than initiated reflexively.

### 7.3. A Case-Based Clinical Pathway

**Figure 2:**
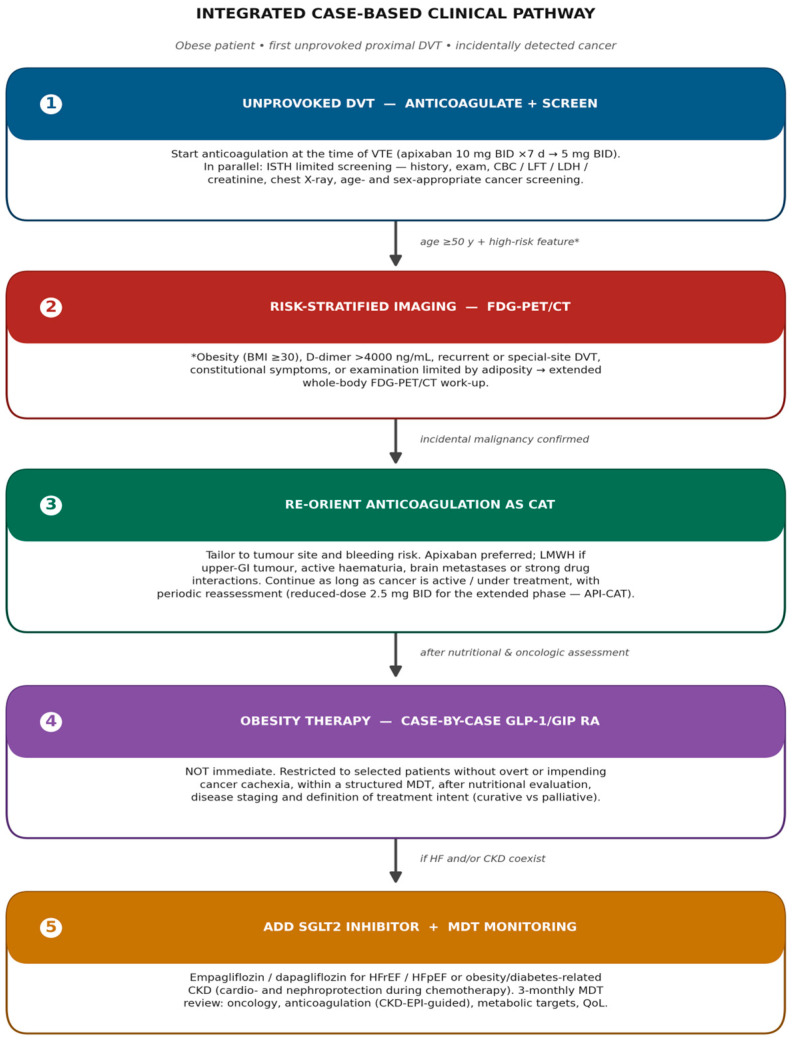
Case-based clinical pathway. Five sequential steps from unprovoked DVT to integrated management, with anticoagulation beginning at the time of venous thromboembolism and being re-oriented—not initiated—by the subsequent cancer diagnosis. Obesity pharmacotherapy is positioned late and gated behind nutritional assessment. * Body composition must also be taken into account in the diagnosis of obesity.

The following vignette (Box 1) anchors the abstract sequence to a concrete decision flow; the corresponding five steps are summarized in Figure 2.

Box 1 **Illustrative clinical vignette.**

A 62-year-old man with obesity (BMI 36 kg/m^2^) with a history of type 2 diabetes presents with a first, unprovoked proximal deep vein thrombosis of the left leg. Therapeutic anticoagulation is started at diagnosis; given the absence of a provoking factor and his age, limited occult-cancer screening is initiated in parallel. Basic laboratory work-up shows a disproportionately elevated D-dimer, and renal function—estimated with CKD-EPI rather than weight-based Cockcroft–Gault—is adequate.

Because he combines age ≥ 50 years, severe obesity that degrades physical examination and cross-sectional imaging, and a high-risk D-dimer, he proceeds to whole-body FDG-PET/CT, which reveals a focal colonic lesion; colonoscopy and histology confirm a colorectal adenocarcinoma. The anticoagulation already in place is now re-framed as cancer-associated thrombosis: apixaban is continued as a fixed-dose oral agent, avoiding weight-based LMWH uncertainty, after confirming the absence of strong CYP3A4/P-gp interactions with his planned oncologic regimen and of a high mucosal-bleeding lesion.

He undergoes surgery and adjuvant chemotherapy; anticoagulation is maintained as long as the cancer is active and under treatment, with periodic reassessment, and reduced-dose apixaban maintenance is considered after the first six months in line with API-CAT. Inherited thrombophilia testing is not performed, as it would not alter management. Obesity pharmacotherapy is deliberately deferred: a GLP-1/GIP receptor agonist such as tirzepatide is considered only later, after nutritional assessment confirms adequate lean-mass reserve and the absence of cancer cachexia, once disease is controlled. An SGLT2 inhibitor is added for cardiorenal protection around potentially cardiotoxic therapy, and the whole plan is managed by an identified treating physician within a multidisciplinary team.

### 7.4. Digital Health and Longitudinal Monitoring

Sustained management of this multimorbid phenotype benefits from structured longitudinal monitoring: body composition and muscle strength to detect incipient sarcopenia before it becomes cachexia, renal function trends to keep anticoagulant dosing safe, and bleeding/thrombosis surveillance synchronized with chemotherapy cycles. Remote and digital tools can support adherence and early detection of deterioration, but they supplement rather than replace periodic multidisciplinary reassessment.

### 7.5. Research Agenda

Several questions remain open. Prospective data are needed on the optimal timing and patient selection for GLP-1/GIP therapy in patients with treated malignancy and adequate nutritional reserve; on whether SGLT2 inhibition confers measurable cardiorenal protection specifically during cardiotoxic cancer therapy in obese patients; and on reduced-dose DOAC strategies in the obese CAT population, who were under-represented in pivotal trials. A dedicated obese-CAT registry capturing body composition, renal-function estimation method, and bleeding outcomes would directly inform the framework proposed here.

## 8. Conclusions

The obese patient who develops unprovoked DVT and proves to harbor an incidental cancer sits at the intersection of metabolic, thrombotic, and oncologic risk, and is best served by an integrated but appropriately sequenced strategy rather than by parallel single-organ management. Anticoagulation begins at the venous thromboembolic event and is re-oriented—not started—by the cancer diagnosis, with apixaban first-line for most patients, attention to CYP3A4/P-gp interactions and renal estimation by CKD-EPI, duration tied to disease activity, and reduced-dose maintenance now supported for selected patients. Obesity pharmacotherapy with GLP-1/GIP receptor agonists carries genuine oncologic promise but must be applied cautiously in active cancer, restricted to carefully selected patients without overt or impending cachexia and preferably within a structured multidisciplinary setting, while SGLT2 inhibitors contribute cardiorenal protection across the cancer-treatment course. Prospective, obesity-specific evidence is required to convert this coherent clinical reasoning into guideline-level recommendations.

## Figures and Tables

**Table 1:** Master table of pivotal randomized and observational evidence underpinning the proposed framework, with the corresponding implication for each domain.

Domain	Trial	Design & Population	Key Result	Implication for Proposed Framework
Occult cancer screening	SOME [9]	RCT; first unprovoked VTE	Adding thoraco-abdomino-pelvic CT to limited screening did not reduce missed cancers (Δ2%, *p* = 0.81)	Limited screening remains the standard first step
	MVTEP [18]	RCT; unprovoked VTE	Negative FDG-PET/CT associated with fewer subsequent cancers (1/186 vs. 9/193; *p* = 0.02)	FDG-PET/CT reserved for selected high-risk patients
Obesity/metabolic treatment	SELECT (semaglutide) [19]	RCT; 17,604 overweight/obese with CVD, no diabetes	Semaglutide 2.4 mg ↓ MACE 20% (HR 0.80; 95% CI 0.72–0.90); hsCRP ↓ ≈40%	GLP-1RA preferred where cardiovascular risk is high
	SURMOUNT-1 (tirzepatide) [20]	RCT; 2539 obesity, no diabetes	Tirzepatide 15 mg: −20.9% body weight at 72 weeks	Dual GLP-1/GIP agonist highly effective for weight reduction
	SURPASS-2 (tirzepatide) [21]	RCT; 1879 type 2 diabetes	Tirzepatide superior to semaglutide 1 mg for HbA1c and weight	Supports twincretin efficacy when diabetes coexists
	JAMA Oncology cohort 2025 [22]	Retrospective cohort; 86,632 adults with overweight/obesity	GLP-1RA use associated with 17% lower overall cancer incidence (HR 0.83; 95% CI 0.76–0.91)	Reassuring—though not antineoplastic—oncologic safety profile
Cardiorenal protection (SGLT2 inhibitors)	EMPA-REG OUTCOME (empagliflozin) [23]	RCT; type 2 diabetes + CVD	↓ CV death 38%, ↓ HF hospitalization 35%, renal benefit	Establishes cardiorenal role of SGLT2 inhibitors during cancer therapy
	DAPA-HF (dapagliflozin) [24]	RCT; HFrEF, with/without diabetes	↓ CV death/worsening heart failure	Extends cardioprotection to HF independent of diabetes
	DAPA-CKD (dapagliflozin) [25]	RCT; CKD, with/without diabetes	↓ sustained eGFR decline, ESKD, CV death	Supports renal protection during cancer therapy
Cancer-associated thrombosis (CAT)	HOKUSAI-VTE Cancer (edoxaban) [26]	RCT; CAT	Noninferior to dalteparin; higher major GI bleeding	Early DOAC evidence; GI-bleeding signal informs agent selection
	SELECT-D (rivaroxaban) [27]	RCT; CAT	↓ 6-month recurrence (4% vs. 11%); more CRNMB	Confirms DOAC efficacy with a bleeding trade-off
	CARAVAGGIO (apixaban) [28]	RCT; 1170 CAT	Noninferior to dalteparin; no excess upper-GI bleeding	Preferred DOAC for established CAT
	AVERT—obesity subgroup (apixaban) [29]	Primary thromboprophylaxis; ambulatory cancer on chemotherapy	↓ VTE in obese (HR 0.26) and non-obese (HR 0.54); not a CAT-treatment trial	Prophylactic—not CAT-treatment—efficacy consistent across BMI strata
	API-CAT (apixaban) [30]	RCT; 1766 active cancer, extended phase	Reduced-dose 2.5 mg BID noninferior to 5 mg BID; less clinically relevant bleeding	Supports reduced-dose extended-phase strategy (see body-weight caveat, Section 6.6)

BID, twice daily; BMI, body mass index; CAT, cancer-associated thrombosis; CKD, chronic kidney disease; CRNMB, clinically relevant non-major bleeding; CT, computed tomography; CV, cardiovascular; CVD, cardiovascular disease; DOAC, direct oral anticoagulant; eGFR, estimated glomerular filtration rate; ESKD, end-stage kidney disease; FDG-PET/CT, [^18^F]fluorodeoxyglucose positron-emission tomography/computed tomography; GI, gastrointestinal; GLP-1/GIP, glucagon-like peptide-1/glucose-dependent insulinotropic polypeptide; GLP-1RA, GLP-1 receptor agonist; HF, heart failure; HFrEF, heart failure with reduced ejection fraction; hsCRP, high-sensitivity C-reactive protein; MACE, major adverse cardiovascular events; RCT, randomized controlled trial; VTE, venous thromboembolism. The AVERT entry refers to primary thromboprophylaxis, not to treatment of established CAT. ↓, decrease.

**Table 2:** Representative interactions between apixaban and anticancer therapy. Mechanistic categories with concrete examples; the list is illustrative, not exhaustive. AR, androgen receptor; P-gp, P-glycoprotein.

Interaction Class	Representative Anticancer Agents	Net Effect/Action
Strong CYP3A4 + P-gp inducers	Enzalutamide, apalutamide (AR inhibitors); dexamethasone	↓ Apixaban exposure: thrombotic risk; prefer LMWH or avoid combination
Strong CYP3A4 + P-gp inhibitors	Itraconazole, posaconazole; ritonavir; idelalisib	↑ Apixaban exposure: bleeding risk; prefer LMWH or avoid combination
Mucosal/vascular bleeding risk	Bevacizumab and other VEGF inhibitors	↑ Mucosal bleeding independent of PK; reassess agent and intensity

↓, decreasing trend; ↑, increasing trend;

## Data Availability

No new data were created or analyzed in this study. Data sharing is not applicable to this article.
